# The WSTF-ISWI Chromatin Remodeling Complex Transiently Associates with the Human Inactive X Chromosome during Late S-Phase Prior to BRCA1 and γ-H2AX

**DOI:** 10.1371/journal.pone.0050023

**Published:** 2012-11-14

**Authors:** Ashley E. Culver-Cochran, Brian P. Chadwick

**Affiliations:** Department of Biological Science, Florida State University, Tallahassee, Florida, United States of America; St. Georges University of London, United Kingdom

## Abstract

Replicating the genome prior to each somatic cell division not only requires precise duplication of the genetic information, but also accurately reestablishing the epigenetic signatures that instruct how the genetic material is to be interpreted in the daughter cells. The mammalian inactive X chromosome (Xi), which is faithfully inherited in a silent state in each daughter cell, provides an excellent model of epigenetic regulation. While much is known about the early stages of X chromosome inactivation, much less is understood with regards to retaining the Xi chromatin through somatic cell division. Here we report that the WSTF-ISWI chromatin remodeling complex (WICH) associates with the Xi during late S-phase as the Xi DNA is replicated. Elevated levels of WICH at the Xi is restricted to late S-phase and appears before BRCA1 and **γ**-H2A.X. The sequential appearance of WICH and BRCA1/γ-H2A.X implicate each as performing important but distinct roles in the maturation and maintenance of heterochromatin at the Xi.

## Introduction

During each somatic cell division, DNA of the parent cell is precisely duplicated and distributed into two identical daughter cells. As a result of this process, however, more than just genetic information is inherited. The packaging of the DNA as heterochromatin and euchromatin must also be faithfully reestablished in order to maintain the epigenetic state of the genome, or the epigenome [1]. Failure to successfully reinstitute the epigenetic signature is a common feature of disease [2]. Alteration of the epigenome to switch genes on or off is critical for the differentiation of cells in order to permit highly specialized function. To achieve gene silencing throughout the developmental process, facultative heterochromatin must be established and maintained. The most extensive example of facultative heterochromatin is the mammalian inactive X chromosome (Xi), the end product of X chromosome inactivation (XCI)[3], an essential process that balances the levels of X-linked gene expression between the sexes by silencing most gene expression chromosome-wide [4], [5]. Consequently, due to its size the Xi can be readily detected in female nuclei [6], providing a valuable cytological model to investigate facultative heterochromatin in general.

In eutherian mammals, an integral component of the process is the X-inactive specific transcript (XIST). XIST is a long non-coding RNA that is expressed exclusively from the chosen Xi [7], [8], and remains physically associated with the territory of the chromosome at interphase [9]. XIST RNA recruits various chromatin complexes to the Xi [10] that assist in repackaging the chromosome into facultative heterochromatin [11] characterized by increased CpG methylation [12], [13], acquisition of repressive histone modifications/variants [14]–[20], and a substantial delay in DNA replication during S-phase relative to the active X chromosome (Xa) [21], [22].

Once established, the Xi is remarkably stable and remains silent even in the absence of XIST/Xist [23], [24], [25]. Many investigations probing the role of chromatin features in maintaining gene silencing at the Xi have taken advantage of a mouse X-chromosome integrated green fluorescent protein (GFP) transgene [26], that is silenced when located on the Xi. Reactivation from the Xi can be measured by the appearance of GFP. However, the number of cells showing reactivation is very low [24], [27], [28], [29], even when multiple chromatin features are disrupted simultaneously [30]. In fact, global reactivation has only been achieved through fusion of female mammalian cells with mouse embryonal carcinoma cells [31]–[35], or through forced expression in mouse of exogenous factors as cells revert to a pluripotent state [36]. These observations highlight the stability of facultative heterochromatin at the Xi.

**Figure 1:**
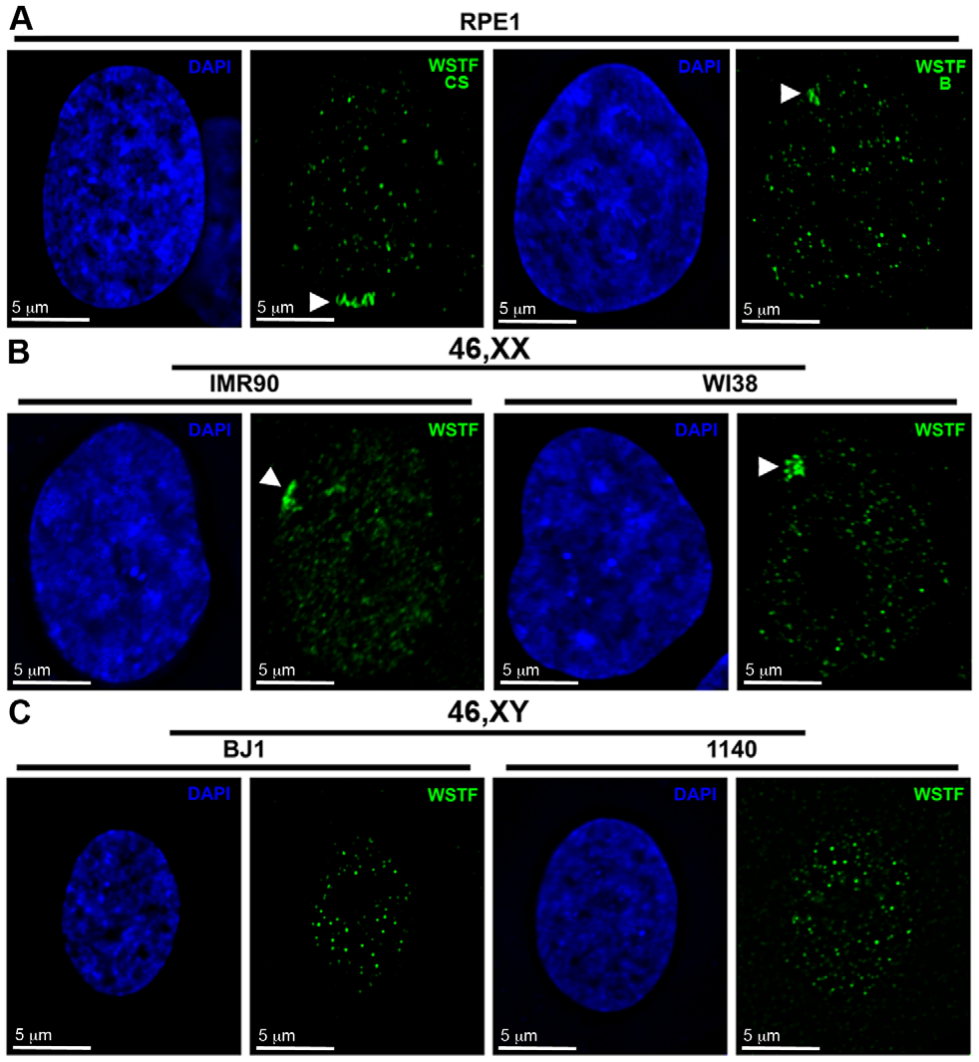
WSTF is enriched in the vicinity of the Barr body in female nuclei with no comparable region of enrichment in male nuclei. **A** Representative hTERT-RPE1 46,XX nuclei showing WSTF enrichment by indirect immunofluorescence with anti-WSTF from Cell Signaling Technology (CS; Green, left image) or anti-WSTF from Bethyl Laboratories (B; Green, right image). White arrowheads point to the area of enrichment. Nuclei counterstained with DAPI (Blue). **B** Representative female (top row; IMR90 and WI38) and male (bottom row, hTERT-BJ1 and 1140) nuclei showing WSTF distribution (Cell Signaling anti-WSTF, green). White arrowheads point to areas of enrichment. Nuclei counterstained with DAPI (Blue).

**Table 1:** Frequency of WSTF enrichment at the Xi.

Cell Type	Number of nuclei scored	Number of nuclei enriched for WSTF at Xi (%)
RPE1 (46,XX)	2005	37 (1.85)
IMR90 (46,XX)	596	5 (0.84)
WI38 (46,XX)	899	9 (1.00)
BJ1 (46,XY)	321	0 (0.00)
1140 (46,XY)	357	0 (0.00)

Although much is known about the initiation of XCI and the resultant chromatin changes [10], our understanding of how the Xi chromatin is faithfully maintained throughout each cell cycle is limited. An attractive period in which facultative heterochromatin can be maintained is during S-phase as the underlying DNA sequence is replicated [37]. The Xi replicates in late S-phase [21], [22], and as such one would anticipate transient association of chromatin complexes with the Xi during this period of the cell cycle. We have previously identified the Breast Cancer 1 gene (BRCA1) and the histone variant H2A.X phosphorylated at serine 139 (γ-H2A.X) as features that transiently appear at the Xi during its replication in late S-phase [38]. BRCA1 is a multifunctional nuclear phosphoprotein [39] that has a central role in maintaining genome integrity as part of the DNA damage response pathway [40]. Other functions of BRCA1 include associating with a chromatin-remodeling complex [41], inducing alterations in chromatin condensation [42], and assisting in heterochromatin mediated gene silencing via ubiquitylation of histone H2A [43]. The histone variant H2A.X is phosphorylated in response to DNA damage [44] and is common to several different DNA damage response pathways throughout the cell cycle [45], including modulating homology-directed double strand break repair with BRCA1 during S-phase [46]. Although the nature of the relationship between BRCA1 and the Xi remains unresolved [47], [48], many functional attributes of the protein support a potential role for BRCA1 in maintaining heterochromatin at the Xi during late S-phase [28].

**Figure 2:**
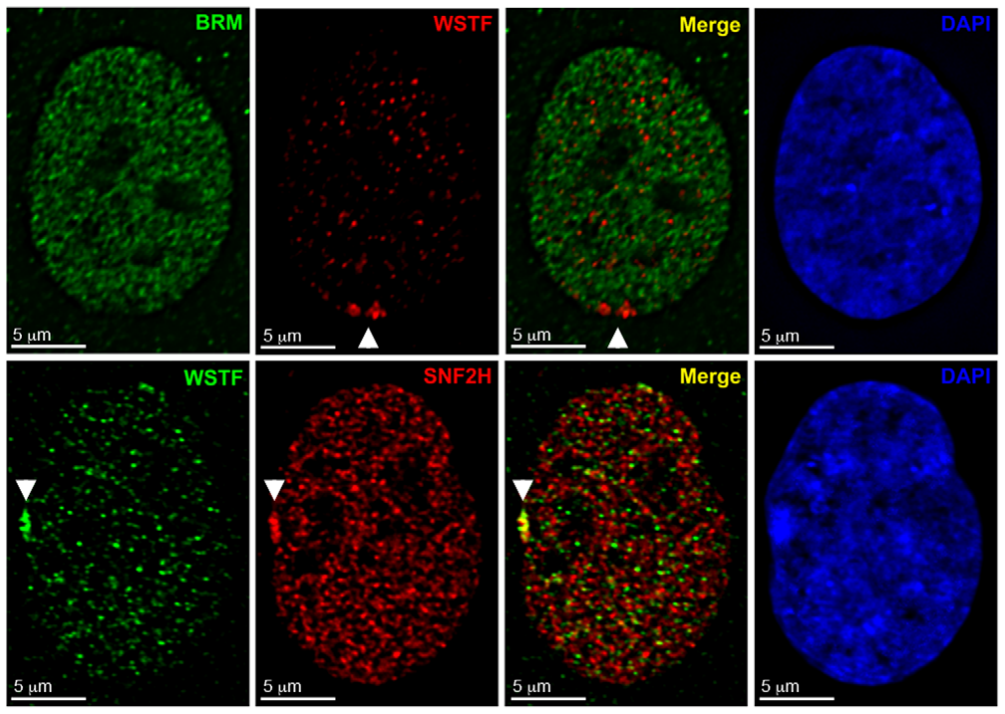
WSTF associates with the Xi as part of either WICH or B-WICH and not the WINAC complex. Top row: Indirect immunofluorescent image of hTERT-RPE1 nucleus showing the distribution of BRM (Green) and WSTF (Red). Bottom row: Indirect immunofluorescent image of hTERT-RPE1 nucleus showing distribution of WSTF (Green) and SNF2H (Red). Overlapping signals appear yellow. White arrowheads point to the Xi. Nuclei are counterstained with DAPI (Blue).

Alternatively, it is also possible that BRCA1 and γ-H2A.X are acting at the Xi during late S-phase in response to DNA damage incurred during replication [46]. Replication forks may stall or collapse when impeded, such as when secondary structure, transcription machinery, or a replication fork barrier is encountered [49]. Recent studies have highlighted that the DNA damage response and repair pathways not only rectify damage generated by the replication process, but also regulate the progression of replication forks [50], [51].

**Figure 3:**
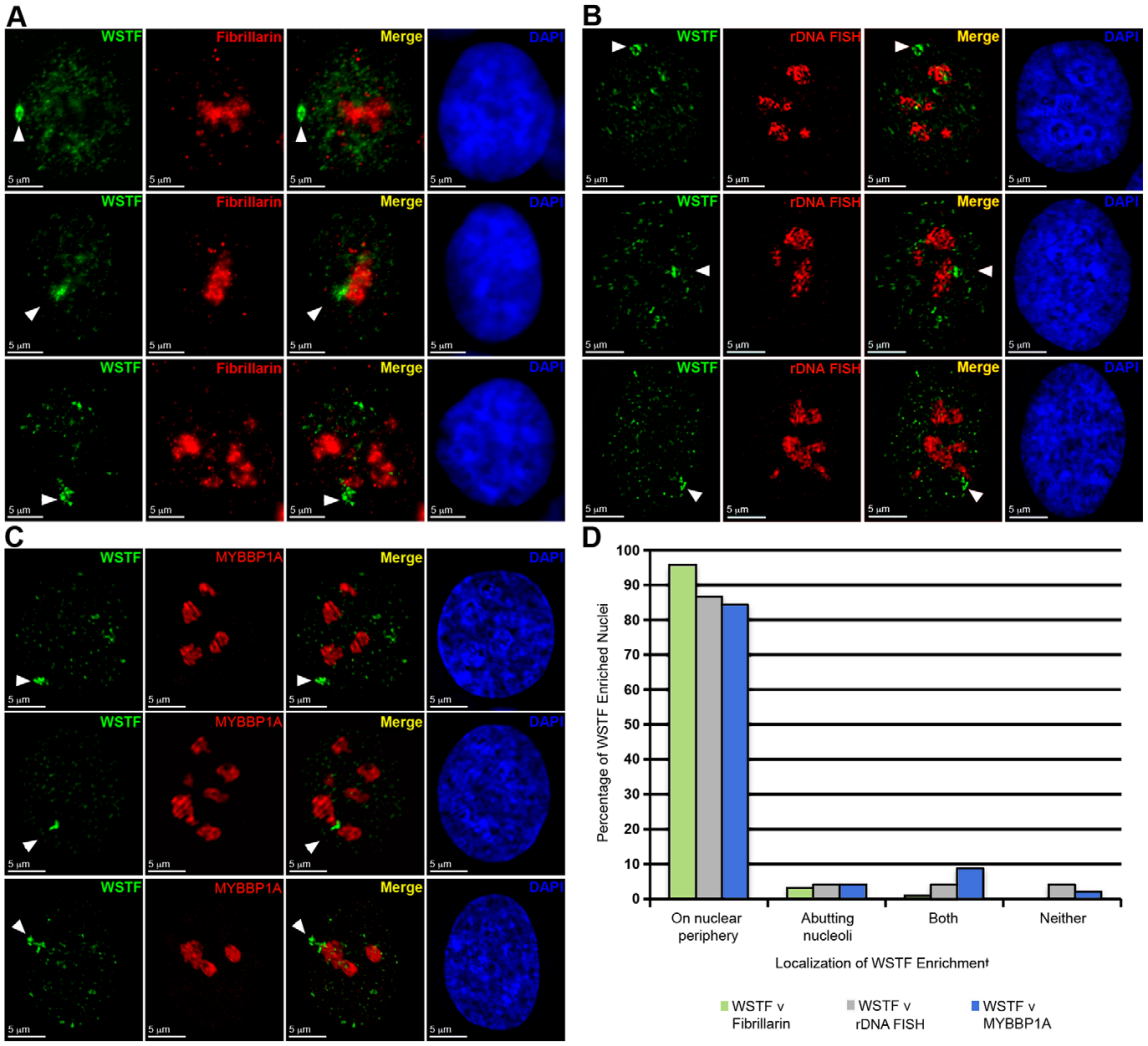
WSTF associates with the Xi in the WICH complex and is most frequently observed at the nuclear periphery and not in the vicinity of nucleoli. **A** hTERT-RPE1 nuclei showing WSTF enrichment (Green) and the location of nucleoli based on Fibrillarin distribution (Red) by indirect immunofluorescence. Examples show WSTF enrichment at the nuclear periphery (Top row), abutting a nucleoli (Middle row), and both peripheral and adjacent to a nucleoli (Bottom row). White arrowheads point to the Xi. Nuclei counterstained with DAPI (Blue). **B** Representative examples, as above, of hTERT-RPE1 nuclei showing WSTF enrichment (Green) and the location of nucleoli based on the hybridization pattern of a direct-labeled rDNA PAC probe RP5-1174A5 (Red). **C** Representative examples, as above, of hTERT-RPE1 nuclei showing WSTF enrichment (Green) and the location of nucleoli based on MYBBP1A distribution (Red) by indirect immunofluorescence. **D** Graph showing the percentage of nuclei for which the location of WSTF enrichment was comparable to one of the three examples depicted in parts **A** (green, n = 93 from five independent experiments), **B** (gray, n = 46 from two independent experiments), and **C** (blue, n = 45 from two independent experiments).

The goal of this study is to examine the attributes of the protein termed Williams syndrome transcription factor (WSTF)[52], [53], which has ties to both chromatin remodeling [54] and in mediating the DNA damage response [55]. Here we report the transient association of WSTF partnered with the ATPase subunit sucrose nonfermenting homolog (SNF2H) in the WSTF-ISWI Chromatin remodeling complex (WICH)[56] with the Xi during late S-phase as the chromosome is replicated. WSTF is encoded by the WBSCR9 gene at chromosome 7q11.23, and is one of approximately 28 genes that are lost due to a large deletion of 1.5–1.8 Mb from one copy of chromosome 7 in Williams syndrome (OMIM#194050)[57]. WSTF is a versatile nuclear protein that is a central component of several different chromatin remodeling complexes [58], and more recently was shown to be the kinase responsible for tyrosine 142 phosphorylation of H2A.X [55], a modification that modulates the H2A.X-mediated DNA damage response pathway. Like BRCA1, the many functional attributes of WSTF suggest that this protein may play a multifaceted role at newly replicated DNA.

**Figure 4:**
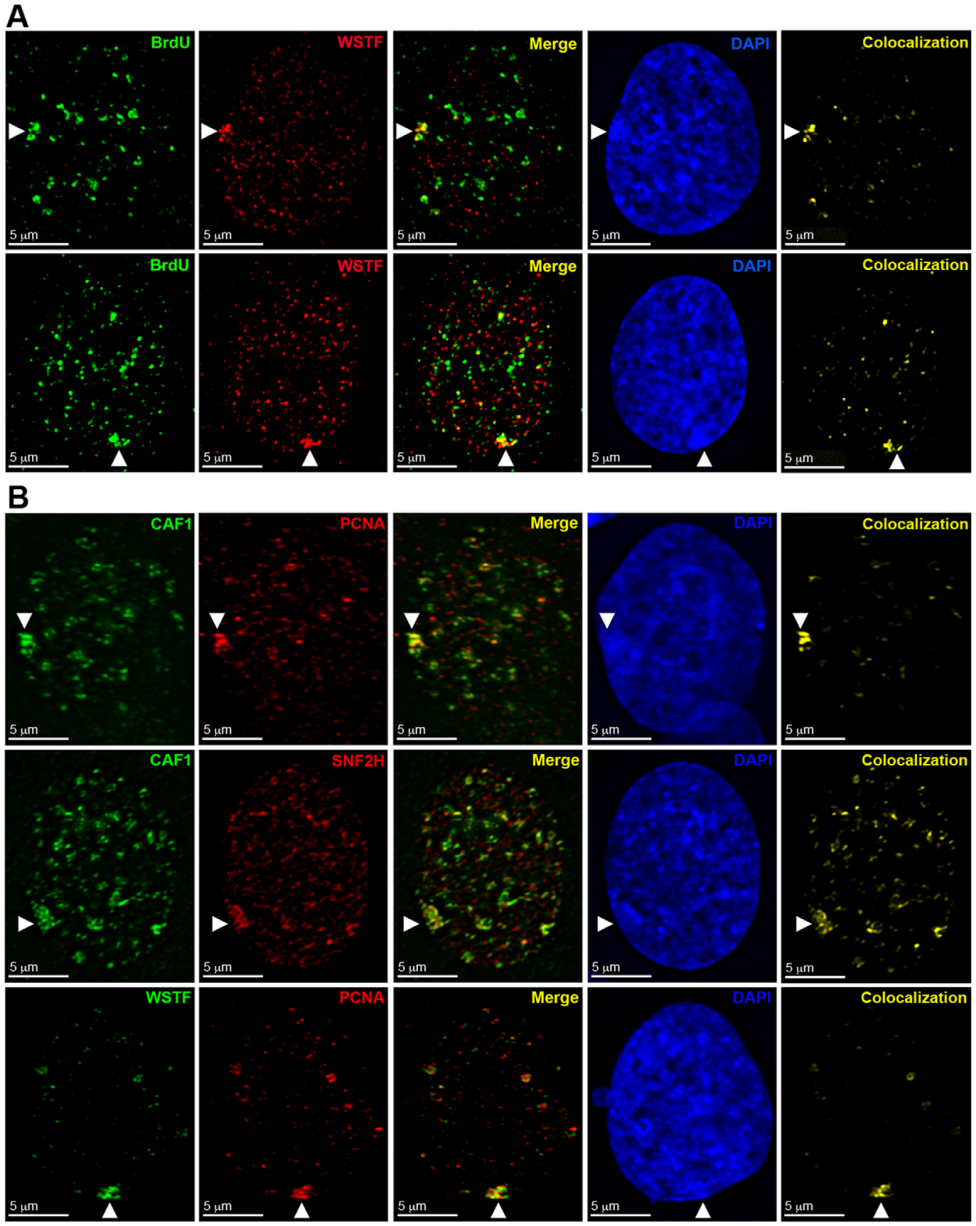
The WICH complex is enriched at the Xi during late S-phase as the Xi is replicated. **A** hTERT-RPE1 cells showing the distribution of WSTF (Red) by indirect immunofluorescence relative to DNA replicated in the last 10 minutes indicated by BrdU incorporation detected by direct immunofluorescence (Green). White arrowheads point to the Xi. Nuclei counterstained with DAPI (Blue). The last panel in the series highlights the overlap between WSTF and BrdU (Yellow). **B** WSTF and SNF2H enrichment relative to the replication fork and sites of chromatin assembly, designated by PCNA and CAF1. Images show examples of indirect immunofluorescence in hTERT-RPE1 nuclei. Top row: CAF1 (Green) and PCNA (Red). Middle row: CAF1 (Green) and SNF2H (Red). Bottom row: WSTF (Green) and PCNA (Red). White arrowheads point to the Xi. Nuclei counterstained with DAPI (Blue). The last panel in the series highlights the overlap between the red and green channels (Yellow).

## Materials and Methods

### Cell culture and BrdU labeling

Cell lines used include: hTERT-RPE1, a 46,XX telomerase-immortalized human cell line derived from the retinal pigment epithelial primary cells RPE-340 (Clontech, Laboratories, Inc., No. C4000-1), hTERT-BJ1, a 46,XY telomerase immortalized human cell line derived from a primary foreskin primary fibroblast cells (Clontech Laboratories, Inc., No C4001-1), 46,XY primary skin fibroblasts CCD-1140sk (ATCC CRL-2714), and 46,XX primary lung fibroblast cells IMR90 (ATCC CCL-186) and WI38 (ATCC CCL-85). All cells were maintained according to the supplier's recommendations. Incorporation of 5-Bromo-2′-deoxyuridine (BrdU; Sigma, B5002) into DNA was achieved by supplementing culture media with 40μM BrdU for 10 min prior to fixing and extracting cells as described below.

**Figure 5:**
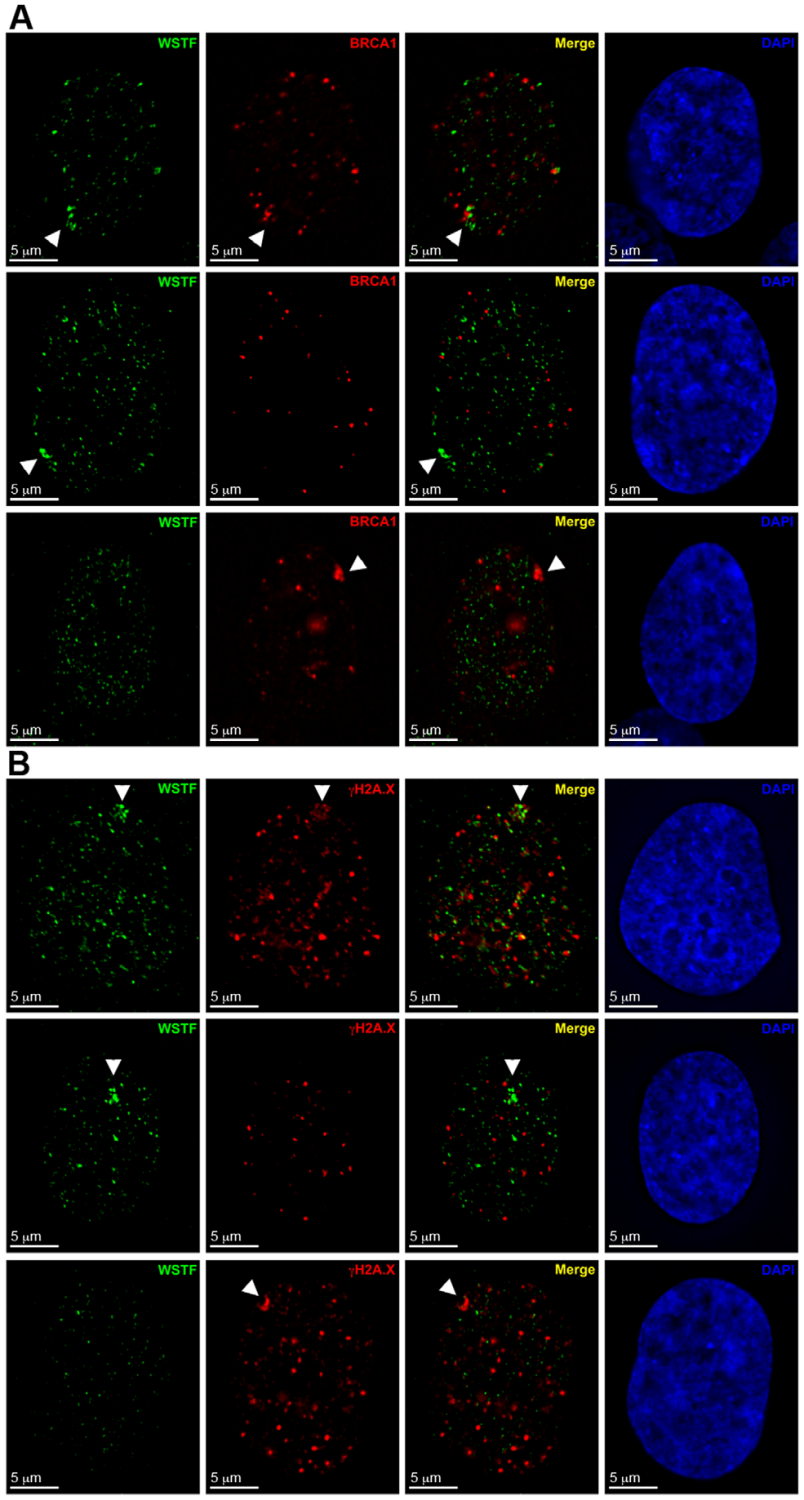
WICH enrichment at the Xi is spatially and temporally distinct from BRCA1 and γ-H2A.X association. **A** Examples of hTERT-RPE1 nucleus showing the distribution by indirect immunofluorescence of WSTF (Green) and BRCA1 (Red). Nuclei counterstained with DAPI (Blue). White arrowheads point to the Xi. Top row: WSTF enrichment with BRCA1 enrichment. Middle row: WSTF enrichment without BRCA1 enrichment. Bottom row: both BRCA1 enrichment without WSTF. **B** Representative examples as above, but showing γ-H2A.X (Red) instead of BRCA1. White arrowheads point to the Xi.

### Antibodies

Rabbit anti-WSTF antibodies were obtained from Cell Signaling Technology (Cat No. 2152) and Bethyl Laboratories (Cat No. A300–446A). Mouse monoclonal antibodies to Fibrillarin (Cat No. ab4566–250) were obtained from Abcam. Anti-BrdU-FITC (Cat No. 1–202–693) antibodies were obtained from Roche. Goat polyclonal anti-BRM antibodies (Cat No. sc-6450), rabbit anti-CAF-1 antibodies (Cat No. sc-10772), and mouse monoclonal antibodies to PCNA (Cat No. sc-56) and BRCA1 (Cat No. sc-6954) were all obtained from Santa Cruz Biotech. Mouse monoclonal antibodies to phospho-H2A.X (Serine-139)(Cat No. 05–636) and SNF2H (Cat No. 05–698) were obtained from Millipore, as were rabbit polyclonal antibodies to histone H3 trimethylated at lysine-27 (Cat No. 07–449) histone H3 phosphorylated at serine 10 (Cat No. 06–570), histone H3 dimethylated at lysine 4 (Cat No. 07–0030), histone H3 acetylated at lysine 9 (Cat No. 06–942), and histone H3 trimethylated at lysine 9 (Cat No. 07–523). Mouse anti-HP1α (Cat No. MAB3584), anti-HP1β (Cat No. MAB3448), and anti-HP1γ (Cat No. MAB3450) were obtained from Chemicon International. Mouse anti-MYBBP1A (Cat No. SAB1400390) was obtained from SIGMA-Aldrich. Rabbit anti-macroH2A.1 antibody was described previously [59]. Alexa-Fluor® conjugated secondary antibodies were obtained from Life Technologies Corporation (Invitrogen).

**Figure 6:**
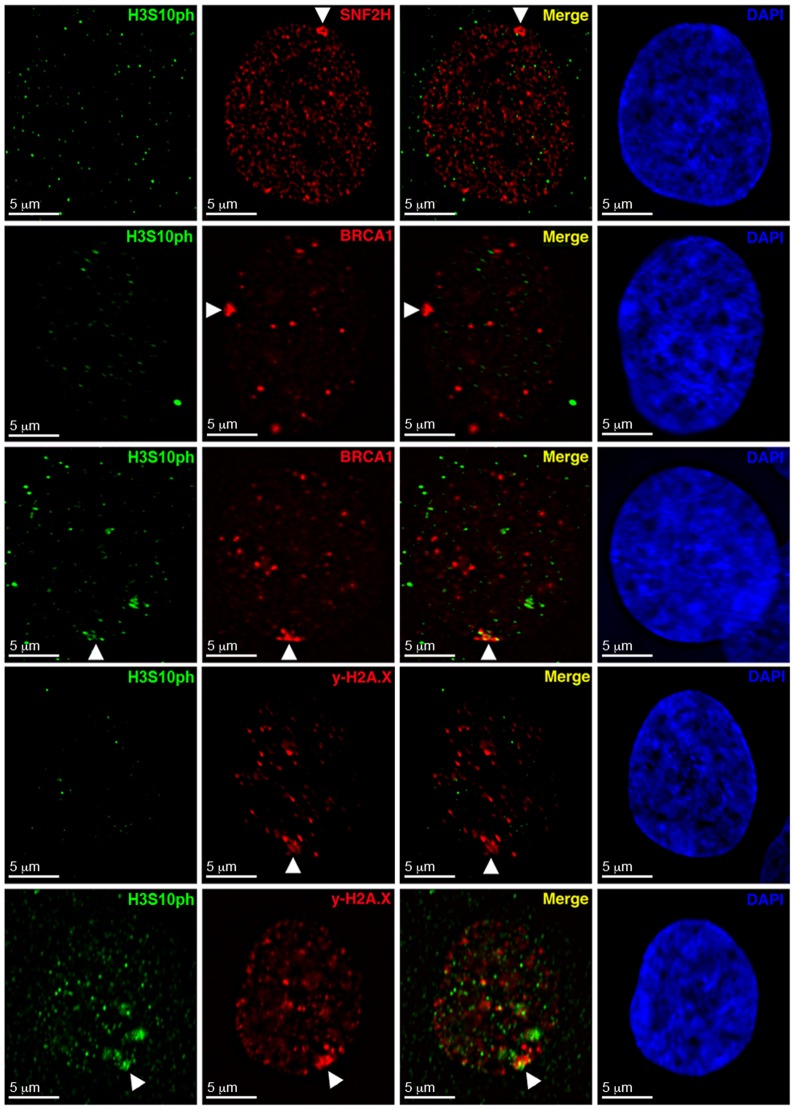
WICH associates with the Xi prior to BRCA1 and γ-H2A.X. Panels show representative images of indirect immunofluorescence to H3S10ph (Green) and SNF2H, BRCA1, or γ-H2A.X (Red) in hTERT-RPE1 nuclei. Top row: SNF2H (Red) and H3S10ph (Green). Second and third rows: BRCA1 (Red) and H3S10ph (Green). Fourth and firth rows: γ-H2A.X (Red) and H3S10ph (Green). Nuclei counterstained with DAPI (Blue). White arrowheads point to the Xi.

**Table 2:** Frequency of the appearance of H3S10ph and/or BrdU incorporation in hTERT-RPE1 cells.

Number of Nuclei	Both H3S10ph and BrdU	H3S10ph only (% of total H3S10ph-containing nuclei)	BrdU only (% of total BrdU-containing nuclei)
237	10	33 (76.74)	34 (77.27)
108	6	20 (76.92)	20 (76.92)

### Immunofluorescence, immuno-DNA FISH, and immuno-RNA FISH

Approximately 200,000 cells were grown directly on slides. Slides were washed once with 1X phosphate buffered saline (PBS) for 2 minutes at room temperature (RT) and fixed in 4% formaldehyde/1X PBS/0.1% Triton X-100 for 10 minutes at RT. Slides were washed twice in 1X PBS for 2 minutes each at RT and blocked in 3% BSA/1X PBS/0.1% Tween 20 for 30 minutes at RT. Slides were washed three times with 1X PBS for 2 minutes each at RT. Slides were incubated with primary antibodies in 1% BSA/1X PBS/0.1% Tween 20 for 1 hour at RT. Slides were washed three times with 1X PBS for 2 minutes each at RT before incubation with secondary antibodies in 1% BSA/1X PBS/0.1% Tween 20 for 1 hour at RT. Slides were washed three times with 1X PBS for 2 minutes each at RT, fixed as above, and finally washed three times with 1X PBS for 2 minutes each at RT. DNA was counterstained using ProLong® Gold antifade reagent supplemented with 4′,6-diamidino-2-phenylindole (DAPI)(Life Technologies Corporation). Detection of BrdU incorporation was performed after staining and fixing. Antibody access was achieved by treating cells in 4N HCl for 10 min at RT prior to neutralizing in 1x PBS and staining with anti-BrdU-FITC for 1 hour in 1%BSA/1xPBS/0.1% Tween 20. Slides were then washed, fixed, and washed again as described above. For immunofluorescence combined with DNA FISH, immunofluorescence was first performed as described above. Bacterial Artificial Chromosomes (BACs) were obtained from the BACPAC resource center (Children's Hospital Oakland Research Institute). P1-artificial chromosome (PAC) RP5-1174A5, which contains rDNA and detects the Nucleolar Organizing Regions (NOR)[60] was obtained from Dr. P. Finelli. The FISH probe was denatured in the presence of human Cot-1 DNA (15279-011, Life Technologies Corporation) at 78°C for 10 minutes followed by 30 minutes at 37°C to block repetitive sequences before applying to the slides. Slides were dehydrated in 70% and 100% ethanol for 3 minutes each at 4°C and then denatured in 50% formamide/2X SSC for 10 minutes at 83°C. Slides were dehydrated once more and air-dried before applying the FISH probe, sealing under cover glass, and incubating overnight at 37°C. Slides were then washed twice in 50% formamide/2X SSC at 43°C for 8 minutes each and once in 2X SSC at 43°C for 8 minutes. Antifade containing DAPI was added to the slides. Immunofluorescence combined with RNA FISH was performed essentially as described [61].

**Figure 7:**
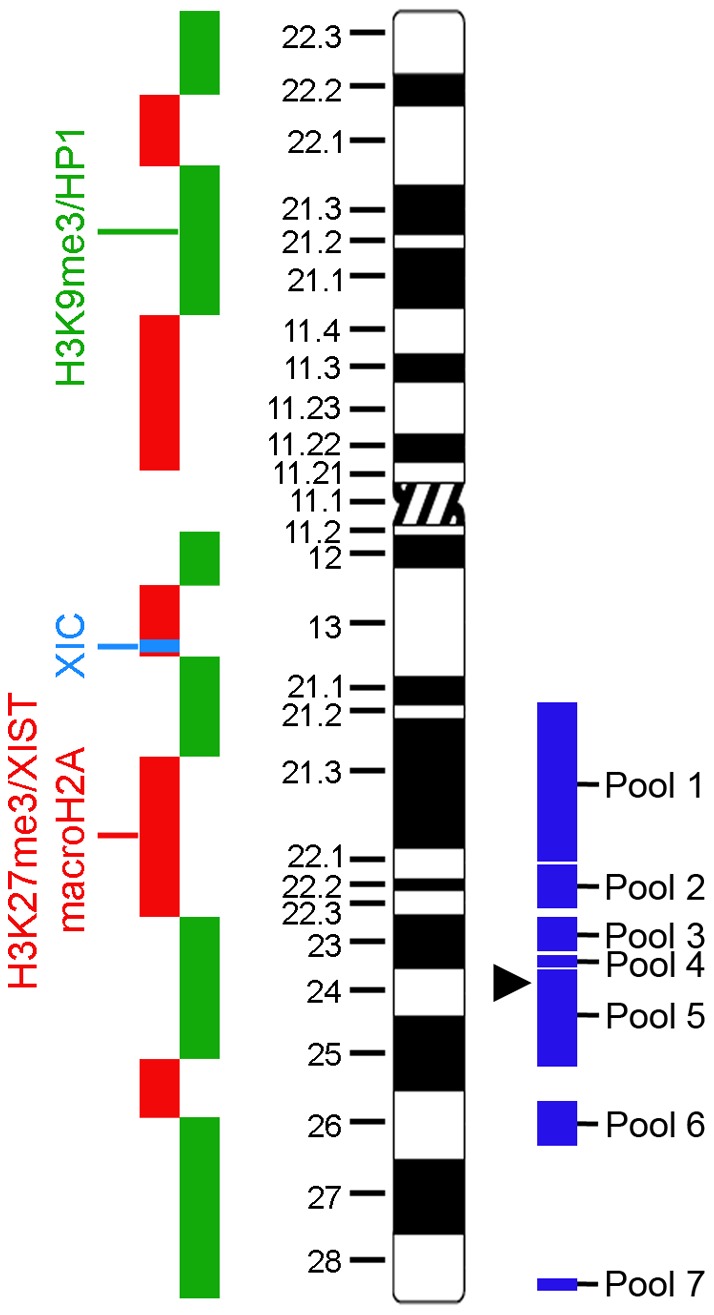
Ideogram of the X chromosome (Center, black and white) detailing the approximate locations of H3K9me3/HP1 (Left, green) and H3K27me3/XIST RNA/macroH2A (Left, red) territories. The X inactivation center (XIC) is depicted in light blue (Left). The groups of BAC clones used are shown as blocks (Right, dark blue) which span the approximate location of all clones in the pool. The location of BAC clone 423G05 is indicated by the black arrowhead. The X chromosome ideogram was adapted from David Adler, University of Washington (www.pathology.washington.edu/research/cytopages/ideograms/human). For a detailed list of the BAC clones, see the Supporting Information, Table S1.

**Figure 8:**
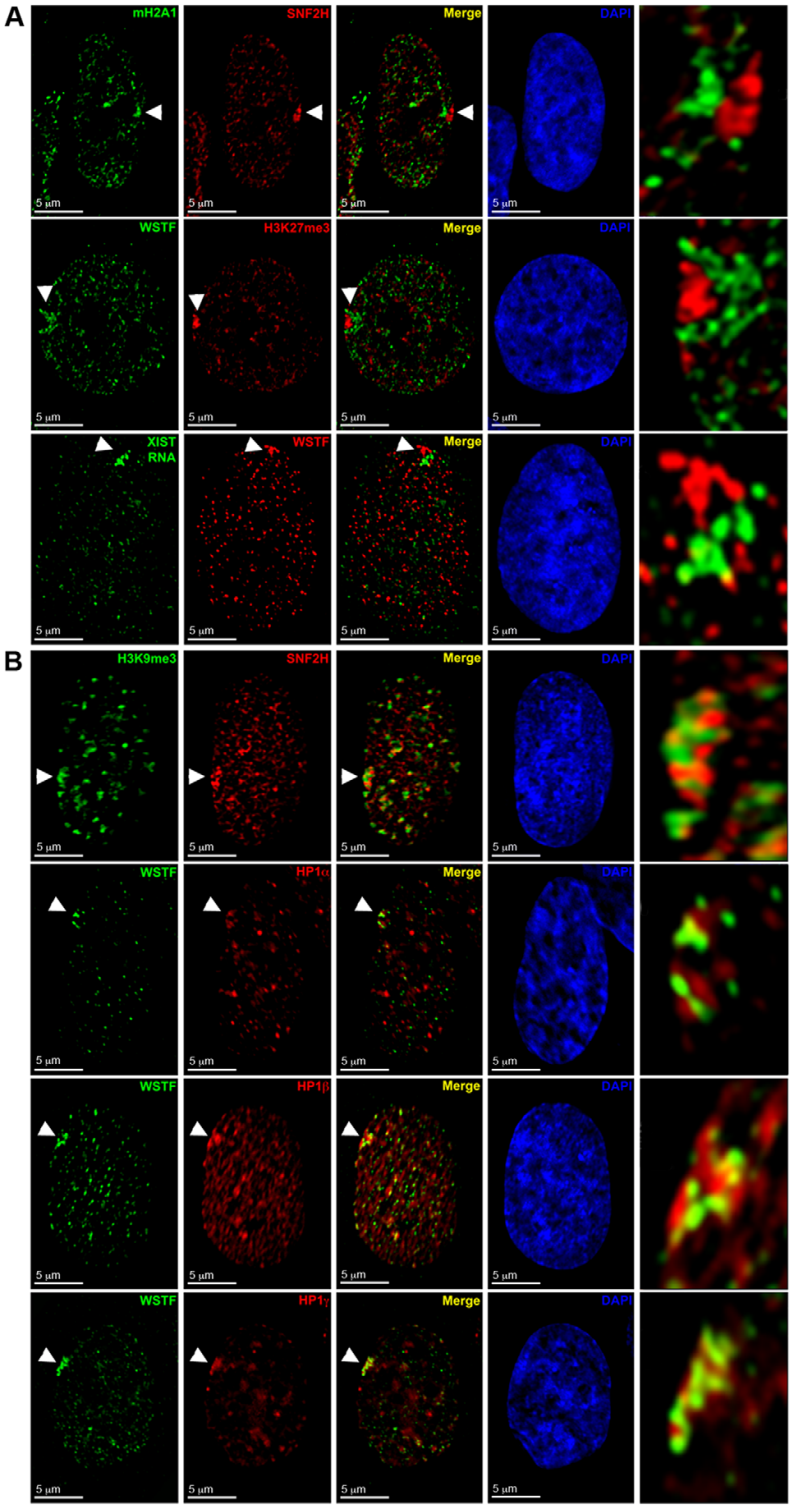
WICH enrichment at the Xi does not overlap with H3K27me3, macroH2A1 or XIST, but does show limited overlap with the HP1 and H3K9me3 territories. **A** WSTF or SNF2H enrichment relative to facultative-like heterochromatin. Images show examples for the distribution of SNF2H (Red) relative to macroH2A.1 (Green, top row), and WSTF (Green) relative to H3K27me3 (Red, middle row) in hTERT-RPE1 nuclei by indirect immunofluorescence. Bottom panels show WSTF distribution by indirect immunofluorescence (Red) relative to hybridization pattern of a direct-labeled XIST probe (Green) in a hTERT-RPE1 nucleus. White arrowheads point to the Xi. Overlapping signals appear yellow. Nuclei counterstained with DAPI (Blue). The last panel in the series shows an enlarged view of the area indicated by white arrowheads. **B** WSTF or SNF2H enrichment relative to constitutive-like heterochromatin marks. Images show examples for the distribution of SNF2H (Red) relative to H3K9me3 (Green, top row) and WSTF (Green) relative to HP1α (Red, second row), HP1β (Red, third row) and HP1γ (Red, bottom row) in hTERT-RPE1 nuclei by indirect immunofluorescence. Overlapping signals appear yellow. Panels to the right show nuclei counterstained with DAPI (Blue). White arrowheads point to the Xi. The last panel in the series shows an enlarged view of the area indicated by white arrowheads.

### Fluorescence *in situ* hybridization probe preparation

Direct-labeled FISH probes were prepared using a Nick Translation kit with SpectrumGreen dUTP according to the manufacturer's recommendations (Abbott Molecular). RP11-423G05 BAC was obtained from the BAC-PAC Resource Center, Children's Hospital Oakland Research Institute.

**Figure 9:**
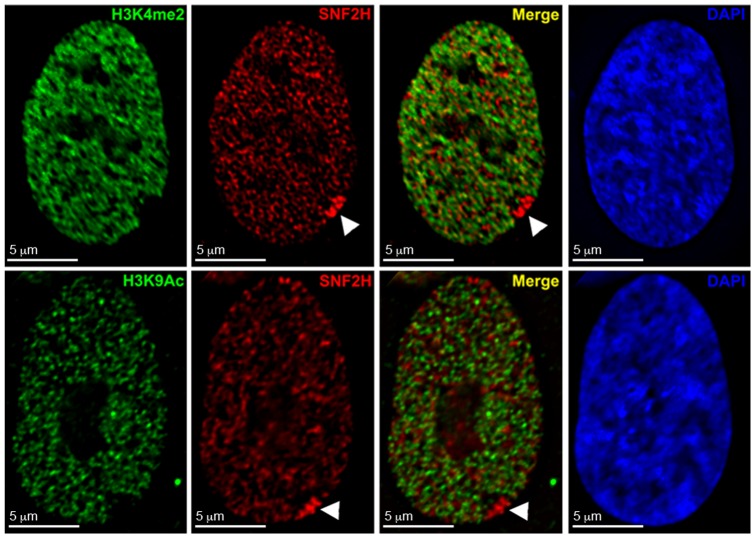
WICH is enriched in the vicinity of the Xi as defined by the hypo-H3K9Ac and hypo-H3K4me2 territory of the Xi at interphase. Representative images showing SNF2H distribution (Red) relative to H3K4me2 (Green, top row) or H3K9Ac (Green, bottom row) in hTERT-RPE1 nuclei by indirect immunofluorescence. White arrowheads point to the Xi. Nuclei were counterstained with DAPI (Blue, far right panels).

**Figure 10:**
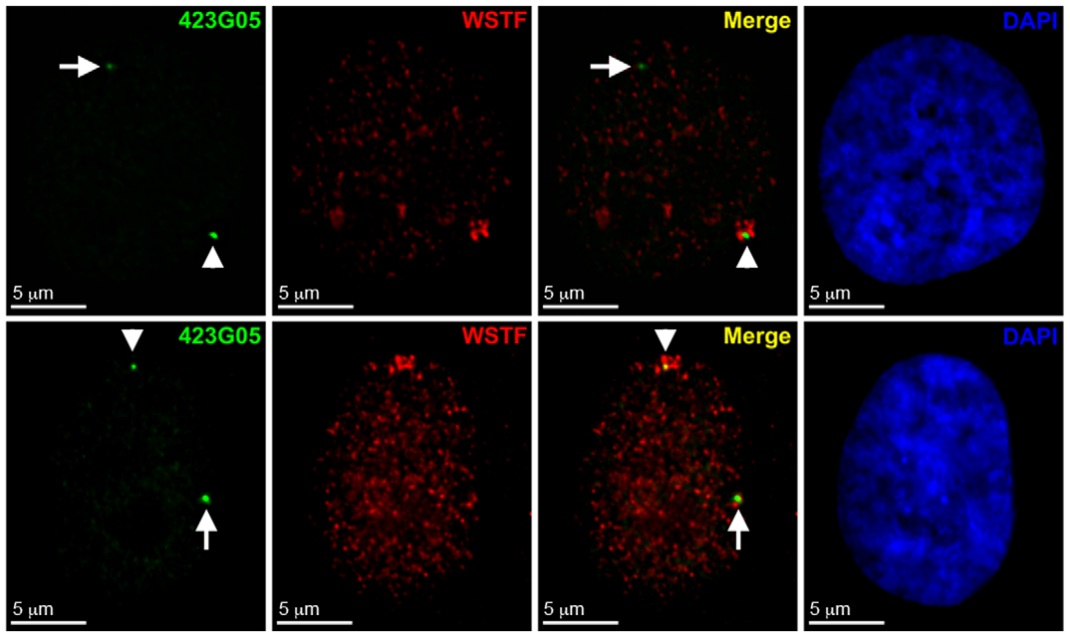
WICH does not associate with all Xi DNA equally as defined by FISH with X-linked BAC probes. Examples show the distribution of WSTF (Red) by indirect immunofluorescence relative to the hybridization pattern of direct-labeled 423G05 BAC probe (Green) in hTERT-RPE1 nuclei. White arrowheads point to the Xi FISH signal, and white arrows point to the FISH signal on the active X (Xa). Nuclei were counterstained with DAPI (Blue).

### Image acquisition

Images were collected using a DeltaVision pDV. Delta Vision images were deconvolved with softWoRx 3.7.0 (Applied Precision) and compiled with Adobe Photoshop CS2 (Adobe Systems).

## Results

### Transient association of WSTF with the human Xi

A previous screen investigating the distribution of a variety of chromatin proteins with the human Xi revealed that most proteins were either enriched, deficient, or showed comparable levels at the Xi relative to their general nuclear distribution [62]. A fourth type of protein, those that are elevated at the Xi in only a small proportion of cells, was also identified. Examination of the nuclear distribution of the multifunctional chromatin protein WSTF [58] in the diploid female hTERT-RPE1 cell line showed that in a small number of cells (Table 1) the protein was enriched in the vicinity of the Barr body (Figure 1A); a densely staining DNA mass that corresponds to the Xi [6]. An indistinguishable pattern of distribution was obtained when using an antibody raised to an independent epitope of WSTF (Compare the left and right panels in Figure 1A) confirming that the enrichment corresponded to WSTF protein and not an antibody artifact. Extending this analysis to other diploid cells indicated that the enrichment was common to all female, but not male cells examined (Figure 1B and Table 1).

### The WICH complex localizes to the Xi

Prior research indicates that WSTF participates in several different complexes [58]. Thus, we sought to determine what context WSTF can be seen in when enrichment is observed at the Xi. Towards this goal, we performed immunostaining on hTERT-RPE1 cells using antibodies raised against WSTF and subunits of the various WSTF-containing complexes. We used BRM for WSTF Including the Nucleosome Assembly Complex (WINAC) [63] and SNF2H [64] for the WICH and B-WICH chromatin remodeling complexes [56], [65].

In hTERT-RPE1 cells, the distribution of WSTF appeared distinct from BRM, and consistent with previous observations, BRM was deficient at the Xi [62] even when WSTF was enriched (Figure 2, top row). In contrast, WSTF showed substantial overlap with SNF2H at the Xi in 100% of cells that had WSTF enrichment (Figure 2, bottom row, n = 72).

WSTF participates in two separate SNF2H-containing complexes. The first complex, WICH, consists of WSTF and SNF2H only [56] and is associated with transcription regulation and chromatin remodeling [54], [56], [66]. The second complex, B-WICH, contains WSTF and SNF2H alongside several other factors [65], and has been linked to ribosomal gene transcription [65], [67].

Since both complexes contain WSTF and SNF2H, the colocalization of these two proteins at the Xi raises the question of whether WSTF enrichment at the Xi occurs as part of the WICH or B-WICH complex. The B-WICH complex has been shown to localize to the fibrillar region of nucleoli in order to participate in both RNA polymerase I and RNA polymerase III transcription [65], [67], [68]. To identify the specific WSTF-complex at the Xi, we performed additional immunofluorescence analyses with antibodies raised against WSTF and the conserved nucleolar protein fibrillarin [69]. Additionally, we performed immuno-DNA FISH using antibodies raised against WSTF and a direct labeled PAC probe, RP5-1174A5, which contains rDNA repeats and highlights the nuclear organizing region (NOR) [60]. Our results indicated that when WSTF enrichment was observed in comparison to fibrillarin, (Figure 3A), 96% of the WSTF enrichments were located on the periphery of nuclei and were not in the vicinity of the fibrillar region of nucleoli (Figure 3D, n = 93). Similarly, in comparison to the NOR regions (Figure 3B), 87% of the WSTF enrichments were not located near a nucleoli, but were found on the nuclear periphery (Figure 3D, n = 46)–evidence that WSTF enrichment at the Xi most likely occurs as part of the WICH complex. To further distinguish WSTF enrichment in the context of the WICH or B-WICH complex, we performed immunofluorescence with antibodies raised against WSTF and a bona fide marker of the B-WICH complex, Myb-binding protein 1A (MYBBP1A)(Figure 3C)[65]. Our results indicated that WSTF enrichment appeared at the nuclear periphery and separate from MYBBP1A in 84% of WSTF enriched nuclei (Figure 3D, n = 45), strongly supporting the conclusion that WSTF enrichment at the Xi is WICH and not B-WICH. From this point forward, where appropriate we will refer to enrichment of SNF2H or WSTF at the Xi as the WICH complex using either as a marker for the chromatin remodeling complex.

### WICH interacts with the Xi during its replication in late S phase

The transient nature of the WICH enrichment at the Xi (Table 1) suggests association in a cell-cycle dependent manner. Prior research has revealed that WSTF associates with pericentric heterochromatin [56] and is recruited to replication foci through interaction with proliferating cell nuclear antigen (PCNA) [54]. Therefore, we sought to determine if elevated levels of WICH at the Xi correlated with late S-phase, the period in which the Xi DNA is replicated [21], [22]. Cells show characteristic patterns of BrdU incorporation for early, mid, and late S-phase [70], and the Xi can be readily detected in late S-phase [71]. Cells were briefly pulse labeled with BrdU and WSTF distribution was examined. WSTF showed substantial, but not complete overlap with BrdU at the Xi (Figure 4A), and all cells with WSTF enrichment at the Xi were in late S-phase (n = 53). Not all late S cells, however, were enriched for WSTF (22.0% of late S-phase cells did not exhibit WSTF enrichment, n = 59) indicating that WICH association with the Xi occurs during a narrow period of late S-phase, accounting for the low number of cells displaying enrichment (Table 1).

To further characterize the relationship of WICH with replicating DNA, we made comparisons to the distribution of the p150 subunit of chromatin assembly factor (CAF1), which plays a critical role in assembly of nucleosomes on newly replicated DNA [72], and the DNA polymerase auxiliary protein PCNA, which coordinates DNA synthesis on the leading and lagging strands [73]. PCNA, along with CAF1, has a central role in the inheritance of chromatin state during S-phase [74] and can be used to highlight the replicating Xi during this point in the cell cycle [38]. These two proteins largely overlapped at the Xi, as can be seen with the example shown in Figure 4B. A comparison of SNF2H or WSTF with CAF1 or PCNA, respectively, showed that while the various protein complexes were apparent at the replicating Xi at the same point in time (Figure 4B), they occupied largely separate territories with relatively small regions of overlap (Figure 4B right panel), and, therefore, some WICH was independently occupying regions of the Xi as it was replicated. Prior studies have shown that WSTF interacts with PCNA [54] and this is believed to be the mechanism used to recruit WICH to replication foci [66]. Our data are mostly consistent with these observations, although sites of WSTF association with the Xi that were not co-occupied by PCNA suggest that WICH can be retained at replicating chromatin in a PCNA-independent manner [54].

### WICH associates with the Xi before BRCA1 and γ-H2A.X

We have previously shown that BRCA1 and γ-H2A.X transiently associate with the Xi during late S-phase [38]. Therefore, we sought to determine the spatiotemporal relationship of WICH with BRCA1 and γ-H2A.X at the Xi by comparing patterns of each with relation to one another as well as to a marker of G2. Although WICH and BRCA1 can be enriched at the Xi at the same time (Figure 5A, top row), the two showed no obvious overlap and instead occupied closely abutting regions of the Xi territory. In 20% of nuclei (n = 65), WSTF enrichment was seen without BRCA1 (Figure 5A, middle row). Likewise, 59.1% of nuclei (n = 44) showed BRCA1 enrichment without WSTF (Figure 5A, bottom row), suggesting a sequential association of the two proteins at the Xi. In contrast, WSTF partially overlapped γ-H2A.X distribution (Figure 5B, top row) in some nuclei, but like BRCA1, nuclei were observed that were enriched only for WSTF (Figure 5B, middle row) or only for γ-H2A.X (Figure 5B, bottom row). Given that BRCA1 also overlaps γ-H2A.X [38] suggests that γ-H2A.X at the Xi bridges the periods of WSTF and BRCA1 association. It remains unknown, however, in what order these proteins associate with the Xi (WICH, γ-H2A.X, then BRCA1 or the opposite order).

Phosphorylation of histone H3 at serine 10 (H3S10ph) initiates at heterochromatic regions during G2 and continues to accumulate as cells proceed toward mitosis [75]. However, in hTERT-RPE1 cells, H3S10ph is detected several hours prior to mitosis [76] with 23.19% of H3S10ph-positive nuclei also exhibiting BrdU incorporation, marking cells in either very late S-phase or G2 (Table 2). Using indirect immunofluorescence, we looked for cells showing elevated levels of SNF2H, BRCA1, and γ-H2A.X at the Xi relative to detection of H3S10ph (Figure 6). SNF2H enrichment never coincided with the appearance of H3S10ph (n = 50). However, H3S10ph was present in 72.4% of BRCA1 enriched nuclei and (n = 76) and 41.5% of γ-H2A.X enriched nuclei (n = 82). We interpret these findings as the WICH complex associating with the Xi prior to both γ-H2A.X and BRCA1, with BRCA1 associating last.

### WICH shows minimal overlap with some but not all chromatin features of the Xi

Heterochromatin of the human Xi is characterized by the *cis* association of XIST RNA [8], the presence of histone variant macroH2A1 [15], binding of the human homologues of drosophila heterochromatin protein 1 (HP1)[62], alpha, beta, and gamma [77], [78], and trimethylation of histone H3 lysine residues 9 (H3K9me3)[79] and 27 (H3K27me3)[19], [20], that are non-randomly arranged into two non-overlapping territories at the Xi (Figure 7) [79]. Previously we reported that BRCA1 and γ-H2A.X do not appear to overlap with chromatin features of the Xi [38]. Similarly, we found that WICH did not overlap with features of the facultative-like heterochromatin on the Xi (macroH2A1, H3K27me3, and XIST RNA; Figure 7, red)[79], but instead occupied comparable sized territories immediately adjacent (Figure 8A). Next we examined the constitutive-like heterochromatin features H3K9me3 and HP1α, HP1β, and HP1γ, thatin hTERT-RPE1 cells occupy a substantial proportion of the Xi [79](Figure 7, green). In most instances these features also appeared distinct from WICH. However, unlike the facultative-like heterochromatin markers that usually occupied territories that were side-by-side with WICH, the constitutive-like heterochromatin markers frequently appeared intertwined with WICH (Figure 8B, right panels). Furthermore, close examination revealed small, but not insignificant regions of overlap with HP1 and H3K9me3–something not observed with macroH2A1, H3K27me3, or XIST.

The limited overlap for some Xi features with WICH and complete lack for others could be interpreted as WICH enrichment reflecting a chromatin territory close to the Xi, but not the Xi itself. To address this, we took two complementary approaches. First, we compared the enrichment of WICH to the distribution of histone H3 dimethylated at lysine 4 (H3K4me2) and H3 acetylated at lysine 9 (H3K9Ac). Nucleosomes of the Xi are hypoacetylated [17], [80] and underrepresented for H3K4me2 [14], which is consistent with the association of both modifications with euchromatin [81]. As such, by immunofluorescence the Xi appears as a distinct hole in the distribution, demarcating the territory of the chromosome [14], [62]. Elevated levels of WICH at the Xi clearly reside within this hypo-H3K9Ac and hypo-H3K4me2 region (Figure 9). Next, we performed WSTF immunofluorescence combined with fluorescence *in situ* hybridization (FISH) with X-linked bacterial artificial chromosomes (BACs). Several BAC clones were selected that reside within bands of macroH2A.1/H3K27me3 or H3K9me3 (Figure 7, blue; Table S1)[82]. While most BACs appeared close to sites of WICH Xi-enrichment (data not shown), one BAC (RP11-423G05) containing the *DOCK11* gene overlapped with WICH enrichment in 74.6% of cells (Figure 10, n = 59; Figure 7, black arrowhead). This suggests that WICH is not associating uniformly with all regions of the Xi, but perhaps its activity is restricted to specific regions.

## Discussion

WSTF has been implicated in heterochromatin replication, in particular HP1-containing heterochromatin [54], [56], and is a candidate for the maintenance of chromatin states in somatic cell division [66]. In a mouse mutagenesis screen, both components of the WICH complex (Snf2h and Wstf) [56] were independently identified as enhancers of transgene variegation [83] supporting a role for this complex in epigenetic inheritance. Here we report that the WICH complex associates with the territory of the Xi as the chromosome is replicated in late S-phase prior to the appearance of γ-H2A.X and BRCA1 at the Xi [38]. While elevated levels of WICH at the Xi appear to precede serine 139 phosphorylation of H2A.X, the two do appear to overlap in some cells. The same is not true for BRCA1 that always appears spatially distinct from WICH and that, with γ-H2A.X, remains associated with the Xi territory into a later point in late S-phase or, possibly, early G2 than WICH enrichment. One interpretation of these data is that phosphorylation of serine 139 of H2A.X occurs at the Xi immediately before BRCA1, which is supported by γ-H2A.X recruiting BRCA1 [84].

Chromatin of the human Xi is composed of two spatially distinct types of heterochromatin for which the underlying DNA replicates during distinct periods of late S-phase [79], [82]. DNA characterized by facultative-like heterochromatin (macroH2A1, H3K27me3, and XIST) replicates in the first part of late S-phase, whereas DNA packaged into constitutive-like heterochromatin (H3K9me3 and HP1) replicates immediately after this period in very late S-phase [79]. Our previous analysis of BRCA1 and γ-H2A.X at the Xi in late S-phase found that neither showed extensive overlap with either heterochromatin type, but instead appeared to reside in a territory immediately adjacent [38], [48]. What role BRCA1 has at the Xi in this capacity remains unclear [28], [47], [48]. Consistent with these observations, we do not see extensive overlap between the WICH complex and features of the Xi. However, close examination of deconvolved images of WSTF with the distribution of HP1 or H3K9me3 does show that at least some regions are overlapping. However, it is important to note that HP1 has been shown to be recruited to sites of DNA damage to assist in repair [85], [86]. It is possible therefore, that when WICH is seen overlapping with HP1 at the Xi, that this HP1 is not necessarily associating with constitutive-like heterochromatin, but is, in fact, localizing to regions of DNA damage, which could be found at either constitutive-like or facultative-like heterochromatin.

Overlap was never seen between WSTF enrichment and the facultative-like heterochromatin type. One plausible explanation for this could be that this type of heterochromatin dissipates as the chromatin is replicated [71] and, as a consequence, the WICH complex could actually be acting on this territory when seen adjacent to HP1 and H3K9me3 chromatin. Alternatively, WICH may not be associating with facultative-like chromatin as suggested by our inability to detect an overlap between WICH and FISH with probes from this territory [82]. Only a probe from the H3K9me3/HP1 region was coincident with WICH enrichment suggesting that its role on the Xi is restricted to the HP1-containing chromatin. Previous WSTF knock-down experiments demonstrated that primarily HP1β-containing regions were impacted by WSTF deficiency [54]. Although we concluded that WSTF enrichment at the human Xi represented the WICH complex and not the nucleolar-associated B-WICH complex, it is interesting that HP1 subtypes have been linked to the transcription of ribosomal genes [87], [88]. Therefore, WSTF may act at HP1β in general, regardless of whether as WICH or B-WICH.

In mouse, replication of the Xi coincides with relocalization of the chromosome to a perinucleolar position, in the vicinity of a region of Snf2h enrichment [89]. It is conceivable that the perinucleolar Snf2h actually represents the WICH complex suggesting a common mechanism of Xi replication between man and mouse. Failure to localize to this region compromised Xi heterochromatin and gene silencing [89], supporting an important role for Snf2h containing complexes in maintaining the Xi. In identifying the WSTF containing complex that is associating with the human Xi, we concluded that it is the WICH complex since WSTF enrichment did not colocalize with a known marker of the nucleoli-associated B-WICH complex [58].

This analysis highlights, however, a difference between human and mouse. Our data indicate that the replicating human Xi with WICH enrichment were found more often at the nuclear periphery than in a perinucleolar region. Nevertheless, the presence of Snf2h/SNF2H is common suggesting that while the subnuclear localization may not be conserved in man, the chromatin-remodeling complex is, and, therefore, is likely important for the maintenance of human Xi chromatin.

WSTF has also been identified as the kinase responsible for the constitutive phosphorylation of H2A.X at tyrosine 142 [55]. Interestingly, the tyrosine 142 phosphorylation is depleted in response to DNA damage by the phosphatase eyes absent 1-3 (EYA1-3)[90]. Removal of this phosphate from tyrosine triggers the recruitment of DNA damage response proteins, such as MDC1 [55], [90], whereas failure to do so activates apoptosis [90]. It is conceivable that elevated levels of WICH at the Xi during late S-phase corresponds to a wave of H2A.X tyrosine 142 phosphorylation, reestablishing the modification in newly formed chromatin. If WICH functions to prime the chromatin for a future DNA damage response, its association at the Xi in this capacity would be unlinked to the presence of γ-H2A.X and recruitment of BRCA1, which would be present after EYA1-3 removed the tyrosine phosphate from H2A.X in response to damage.

Precisely what function is being performed as WICH, γ-H2A.X, and BRCA1 sequentially associate with the Xi is debatable. Given that BRCA1 and WICH are both implicated in the maintenance of heterochromatin [43], [66] and that mouse Xi chromatin is compromised when the kinases responsible for H2A.X serine 139 phosphorylation are inhibited [29] suggests, however, a direct or indirect role in the maintenance of Xi heterochromatin. Additionally, the role of WSTF in reestablishing the ground state for future DNA damage responses [55] and the fact that WSTF associates with newly replicated DNA provides further evidence that the WICH complex is an integral component in chromatin maturation. Further investigation of the function of WICH is required to reveal how this dynamic complex contributes to the many layers of chromatin maturation and maintenance.

## Supporting Information

Table S1 **List of BAC clone pools used for FISH with WSTF indirect-immunofluorescence.** BAC clone members of each pool (1–6 as indicated in Figure 7) are highlighted in different colors and their coordinates on the X (where known) are given based on hg18, as are genes or genomic features of interest in the corresponding BAC. The BAC clone RP11-423G05 that is shown overlapping WSTF in Figure 10 is highlighted in black.
(PDF)

## References

[1] Alabert C, Groth A (2012) Chromatin replication and epigenome maintenance. Nat Rev Mol Cell Biol 13: 153–167. 22358331 10.1038/nrm3288

[2] Portela A, Esteller M (2010) Epigenetic modifications and human disease. Nat Biotechnol 28: 1057–1068. 20944598 10.1038/nbt.1685

[3] Lyon MF (1961) Gene action in the X-chromosome of the mouse (*Mus musculus* L.). Nature 190: 372–373. 13764598 10.1038/190372a0

[4] Carrel L, Willard HF (2005) X-inactivation profile reveals extensive variability in X-linked gene expression in females. Nature 434: 400–404. 15772666 10.1038/nature03479

[5] Yang F, Babak T, Shendure J, Disteche CM (2010) Global survey of escape from X inactivation by RNA-sequencing in mouse. Genome Res 20: 614–622. 20363980 10.1101/gr.103200.109 PMC2860163

[6] Barr ML, Bertram EG (1949) A morphological distinction between neurones of the male and female, and the behaviour of the nucleolar satellite during accelerated nucleoprotein synthesis. Nature 163: 676–677. 18120749 10.1038/163676a0

[7] Brockdorff N, Ashworth A, Kay GF, McCabe VM, Norris DP, et al (1992) The product of the mouse Xist gene is a 15kb inactive X-specific transcript containing no conserved ORF and located in the nucleus. Cell 71: 515–526. 1423610 10.1016/0092-8674(92)90519-i

[8] Brown CJ, Hendrich BD, Rupert JL, Lafreniere RG, Xing Y, et al (1992) The human XIST gene: analysis of a 17 kb inactive X-specific RNA that contains conserved repeats and is highly localized within the nucleus. Cell 71: 527–542. 1423611 10.1016/0092-8674(92)90520-m

[9] Clemson CM, McNeil JA, Willard HF, Lawrence JB (1996) XIST RNA paints the inactive X chromosome at interphase: evidence for a novel RNA involved in nuclear/chromosome structure. J Cell Biol 132: 1–17. 8636206 10.1083/jcb.132.3.259 PMC2120729

[10] Lee JT (2011) Gracefully ageing at 50, X-chromosome inactivation becomes a paradigm for RNA and chromatin control. Nat Rev Mol Cell Biol 12: 815–826. 22108600 10.1038/nrm3231

[11] Wutz A (2011) Gene silencing in X-chromosome inactivation: advances in understanding facultative heterochromatin formation. Nat Rev Genet 12: 542–553. 21765457 10.1038/nrg3035

[12] Mohandas T, Sparkes RS, Shapiro LJ (1981) Reactivation of an inactive human X chromosome: evidence for X inactivation by DNA methylation. Science 211: 393–396. 6164095 10.1126/science.6164095

[13] Pfeifer GP, Tanguay RL, Steigerwald SD, Riggs AD (1990) In vivo footprint and methylation analysis by PCR-aided genomic sequencing: comparison of active and inactive X chromosomal DNA at the CpG island and promoter of human PGK-1. Genes Dev 4: 1277–1287. 2227409 10.1101/gad.4.8.1277

[14] Boggs BA, Cheung P, Heard E, Spector DL, Chinault AC, et al (2002) Differentially methylated forms of histone H3 show unique association patterns with inactive human X chromosomes. Nat Genet 30: 73–76. 11740495 10.1038/ng787

[15] Costanzi C, Pehrson JR (1998) Histone macroH2A1 is concentrated in the inactive X chromosome of female mammals. Nature 393: 599–601. 9634239 10.1038/31275

[16] Heard E, Rougeulle C, Arnaud D, Avner P, Allis CD, et al (2001) Methylation of histone H3 at Lys-9 is an early mark on the X chromosome during X inactivation. Cell 107: 727–738. 11747809 10.1016/s0092-8674(01)00598-0

[17] Jeppesen P, Turner BM (1993) The inactive X chromosome in female mammals is distinguished by a lack of histone H4 acetylation, a cytogenetic marker for gene expression. Cell 74: 281–289. 8343956 10.1016/0092-8674(93)90419-q

[18] Peters AH, Mermoud JE, O'Carroll D, Pagani M, Schweizer D, et al (2002) Histone H3 lysine 9 methylation is an epigenetic imprint of facultative heterochromatin. Nat Genet 30: 77–80. 11740497 10.1038/ng789

[19] Plath K, Fang J, Mlynarczyk-Evans SK, Cao R, Worringer KA, et al (2003) Role of histone H3 lysine 27 methylation in X inactivation. Science 300: 131–135. 12649488 10.1126/science.1084274

[20] Silva J, Mak W, Zvetkova I, Appanah R, Nesterova TB, et al (2003) Establishment of histone h3 methylation on the inactive x chromosome requires transient recruitment of eed-enx1 polycomb group complexes. Dev Cell 4: 481–495. 12689588 10.1016/s1534-5807(03)00068-6

[21] Gilbert CW, Muldal S, Lajthal LG, Rowley J (1962) Time-sequence of human chromosome duplication. Nature 195: 869–873. 13898534 10.1038/195869a0

[22] Morishma A, Grumbach MM, Taylor JH (1962) Asynchronous duplication of human chromosomes and the origin of sex chromatin. Proc Natl Acad Sci USA 48: 756–763. 14476104 10.1073/pnas.48.5.756 PMC220846

[23] Brown CJ, Willard HF (1994) The human X-inactivation centre is not required for maintenance of X-chromosome inactivation. Nature 368: 154–156. 8139659 10.1038/368154a0

[24] Csankovszki G, Panning B, Bates B, Pehrson JR, Jaenisch R (1999) Conditional deletion of Xist disrupts histone macroH2A localization but not maintenance of X inactivation. Nat Genet 22: 323–324. 10431231 10.1038/11887

[25] Rack KA, Chelly J, Gibbons RJ, Rider S, Benjamin D, et al (1994) Absence of the XIST gene from late-replicating isodicentric X chromosomes in leukaemia. Hum Molec Genet 3: 1053–1059. 7981672 10.1093/hmg/3.7.1053

[26] Hadjantonakis AK, Gertsenstein M, Ikawa M, Okabe M, Nagy A (1998) Generating green fluorescent mice by germline transmission of green fluorescent ES cells. Mech Dev 76: 79–90. 9867352 10.1016/s0925-4773(98)00093-8

[27] Chan KM, Zhang H, Malureanu L, van Deursen J, Zhang Z (2011) Diverse factors are involved in maintaining X chromosome inactivation. Proc Natl Acad Sci USA 108: 16699–16704. 21940502 10.1073/pnas.1107616108 PMC3189073

[28] Ganesan S, Silver DP, Greenberg RA, Avni D, Drapkin R, et al (2002) BRCA1 supports XIST RNA concentration on the inactive X chromosome. Cell 111: 393–405. 12419249 10.1016/s0092-8674(02)01052-8

[29] Ouyang Y, Salstrom J, Diaz-Perez S, Nahas S, Matsuno Y, et al (2005) Inhibition of Atm and/or Atr disrupts gene silencing on the inactive X chromosome. Biochem Biophys Res Commun 337: 875–880. 16213462 10.1016/j.bbrc.2005.09.122

[30] Csankovszki G, Nagy A, Jaenisch R (2001) Synergism of Xist RNA, DNA methylation, and histone hypoacetylation in maintaining X chromosome inactivation. J Cell Biol 153: 773–784. 11352938 10.1083/jcb.153.4.773 PMC2192370

[31] Mise N, Sado T, Tada M, Takada S, Takagi N (1996) Activation of the inactive X chromosome induced by cell fusion between a murine EC and female somatic cell accompanies reproducible changes in the methylation pattern of the Xist gene. Exp Cell Res 223: 193–202. 8601395 10.1006/excr.1996.0073

[32] Sado T, Tada T, Takagi N (1996) Mosaic methylation of Xist gene before chromosome inactivation in undifferentiated female mouse embryonic stem and embryonic germ cells. Dev Dyn 205: 421–434. 8901053 10.1002/(SICI)1097-0177(199604)205:4<421::AID-AJA6>3.0.CO;2-K

[33] Takagi N (1983) De novo X-chromosome inactivation in somatic hybrid cells between the XO mouse embryonal carcinoma cell and XY rat lymphocyte. Exp Cell Res 145: 397–404. 6683194 10.1016/0014-4827(83)90018-6

[34] Takagi N (1993) Variable X chromosome inactivation patterns in near-tetraploid murine EC x somatic cell hybrid cells differentiated in vitro. Genetica 88: 107–117. 8224851 10.1007/BF02424467

[35] Takagi N, Yoshida MA, Sugawara O, Sasaki M (1983) Reversal of X-inactivation in female mouse somatic cells hybridized with murine teratocarcinoma stem cells in vitro. Cell 34: 1053–1062. 6627391 10.1016/0092-8674(83)90563-9

[36] Takahashi K, Yamanaka S (2006) Induction of pluripotent stem cells from mouse embryonic and adult fibroblast cultures by defined factors. Cell 126: 663–676. 16904174 10.1016/j.cell.2006.07.024

[37] Probst AV, Dunleavy E, Almouzni G (2009) Epigenetic inheritance during the cell cycle. Nat Rev Mol Cell Biol 10: 192–206. 19234478 10.1038/nrm2640

[38] Chadwick BP, Lane TF (2005) BRCA1 associates with the inactive X chromosome in late S-phase, coupled with transient H2AX phosphorylation. Chromosoma 114: 432–439. 16240122 10.1007/s00412-005-0029-1

[39] Chen Y, Farmer AA, Chen CF, Jones DC, Chen PL, et al (1996) BRCA1 is a 220-kDa nuclear phosphoprotein that is expressed and phosphorylated in a cell cycle-dependent manner. Cancer Res 56: 3168–3172. 8764100

[40] Huen MS, Sy SM, Chen J (2010) BRCA1 and its toolbox for the maintenance of genome integrity. Nat Rev Mol Cell Biol 11: 138–148. 20029420 10.1038/nrm2831 PMC3899800

[41] Bochar DA, Wang L, Beniya H, Kinev A, Xue Y, et al (2000) BRCA1 is associated with a human SWI/SNF-related complex: linking chromatin remodeling to breast cancer. Cell 102: 257–265. 10943845 10.1016/s0092-8674(00)00030-1

[42] Ye Q, Hu YF, Zhong H, Nye AC, Belmont AS, et al (2001) BRCA1-induced large-scale chromatin unfolding and allele-specific effects of cancer-predisposing mutations. J Cell Biol 155: 911–921. 11739404 10.1083/jcb.200108049 PMC2150890

[43] Zhu Q, Pao GM, Huynh AM, Suh H, Tonnu N, et al (2011) BRCA1 tumour suppression occurs via heterochromatin-mediated silencing. Nature 477: 179–184. 21901007 10.1038/nature10371 PMC3240576

[44] Rogakou EP, Pilch DR, Orr AH, Ivanova VS, Bonner WM (1998) DNA double-stranded breaks induce histone H2AX phosphorylation on serine 139. J Biol Chem 273: 5858–5868. 9488723 10.1074/jbc.273.10.5858

[45] Lukas J, Lukas C, Bartek J (2011) More than just a focus: The chromatin response to DNA damage and its role in genome integrity maintenance. Nat Cell Biol 13: 1161–1169. 21968989 10.1038/ncb2344

[46] Yun MH, Hiom K (2009) CtIP-BRCA1 modulates the choice of DNA double-strand-break repair pathway throughout the cell cycle. Nature 459: 460–463. 19357644 10.1038/nature07955 PMC2857324

[47] Pageau GJ, Hall LL, Lawrence JB (2007) BRCA1 does not paint the inactive X to localize XIST RNA but may contribute to broad changes in cancer that impact XIST and Xi heterochromatin. J Cell Biochem 100: 835–850. 17146760 10.1002/jcb.21188

[48] Xiao C, Sharp JA, Kawahara M, Davalos AR, Difilippantonio MJ, et al (2007) The XIST noncoding RNA functions independently of BRCA1 in X inactivation. Cell 128: 977–989. 17350580 10.1016/j.cell.2007.01.034

[49] Jones RM, Petermann E (2012) Replication fork dynamics and the DNA damage response. Biochem J 443: 13–26. 22417748 10.1042/BJ20112100

[50] Garner E, Costanzo V (2009) Studying the DNA damage response using in vitro model systems. DNA Repair (Amst) 8: 1025–1037. 19482562 10.1016/j.dnarep.2009.04.015

[51] Schwab RA, Niedzwiedz W (2011) Visualization of DNA replication in the vertebrate model system DT40 using the DNA fiber technique. J Vis Exp: e3255. 10.3791/3255 PMC3227199 22064662

[52] Lu X, Meng X, Morris CA, Keating MT (1998) A novel human gene, WSTF, is deleted in Williams syndrome. Genomics 54: 241–249. 9828126 10.1006/geno.1998.5578

[53] Peoples RJ, Cisco MJ, Kaplan P, Francke U (1998) Identification of the WBSCR9 gene, encoding a novel transcriptional regulator, in the Williams-Beuren syndrome deletion at 7q11.23. Cytogenet Cell Genet 82: 238–246. 9858827 10.1159/000015110

[54] Poot RA, Bozhenok L, van den Berg DL, Steffensen S, Ferreira F, et al (2004) The Williams syndrome transcription factor interacts with PCNA to target chromatin remodelling by ISWI to replication foci. Nat Cell Biol 6: 1236–1244. 15543136 10.1038/ncb1196

[55] Xiao A, Li H, Shechter D, Ahn SH, Fabrizio LA, et al (2009) WSTF regulates the H2A.X DNA damage response via a novel tyrosine kinase activity. Nature 457: 57–62. 19092802 10.1038/nature07668 PMC2854499

[56] Bozhenok L, Wade PA, Varga-Weisz P (2002) WSTF-ISWI chromatin remodeling complex targets heterochromatic replication foci. Embo J 21: 2231–2241. 11980720 10.1093/emboj/21.9.2231 PMC125993

[57] Schubert C (2009) The genomic basis of the Williams-Beuren syndrome. Cell Mol Life Sci 66: 1178–1197. 19039520 10.1007/s00018-008-8401-y PMC11131529

[58] Barnett C, Krebs JE (2011) WSTF does it all: a multifunctional protein in transcription, repair, and replication. Biochem Cell Biol 89: 12–23. 21326359 10.1139/O10-114 PMC3251257

[59] Chadwick BP, Willard HF (2001) Histone H2A variants and the inactive X chromosome: identification of a second macroH2A variant. Hum Mol Genet 10: 1101–1013. 11331621 10.1093/hmg/10.10.1101

[60] Finelli P, Sirchia SM, Masciadri M, Crippa M, Recalcati MP, et al (2012) Juxtaposition of heterochromatic and euchromatic regions by chromosomal translocation mediates a heterochromatic long-range position effect associated with a severe neurological phenotype. Mol Cyto 5: 16. 10.1186/1755-8166-5-16 PMC3395859 22475481

[61] McLaughlin CR, Chadwick BP (2011) Characterization of DXZ4 conservation in primates implies important functional roles for CTCF binding, array expression and tandem repeat organization on the X chromosome. Genome Biology 12: R37. 21489251 10.1186/gb-2011-12-4-r37 PMC3218863

[62] Chadwick BP, Willard HF (2003) Chromatin of the Barr body: histone and non-histone proteins associated with or excluded from the inactive X chromosome. Hum Mol Genet 12: 2167–2178. 12915472 10.1093/hmg/ddg229

[63] Yoshimura K, Kitagawa H, Fujiki R, Tanabe M, Takezawa S, et al (2009) Distinct function of 2 chromatin remodeling complexes that share a common subunit, Williams syndrome transcription factor (WSTF). Proc Natl Acad Sci USA 106: 9280–9285. 19470456 10.1073/pnas.0901184106 PMC2695106

[64] Lazzaro MA, Picketts DJ (2001) Cloning and characterization of the murine Imitation Switch (ISWI) genes: differential expression patterns suggest distinct developmental roles for Snf2h and Snf2l. J Neurochem 77: 1145–1156. 11359880 10.1046/j.1471-4159.2001.00324.x

[65] Cavellan E, Asp P, Percipalle P, Farrants AK (2006) The WSTF-SNF2h chromatin remodeling complex interacts with several nuclear proteins in transcription. J Biol Chem 281: 16264–16271. 16603771 10.1074/jbc.M600233200

[66] Poot RA, Bozhenok L, van den Berg DL, Hawkes N, Varga-Weisz PD (2005) Chromatin remodeling by WSTF-ISWI at the replication site: opening a window of opportunity for epigenetic inheritance? Cell Cycle 4: 543–546. 15753658 10.4161/cc.4.4.1624

[67] Vintermist A, Bohm S, Sadeghifar F, Louvet E, Mansen A, et al (2011) The chromatin remodelling complex B-WICH changes the chromatin structure and recruits histone acetyl-transferases to active rRNA genes. PLoS ONE 6: e19184. 21559432 10.1371/journal.pone.0019184 PMC3084792

[68] Percipalle P, Fomproix N, Cavellan E, Voit R, Reimer G, et al (2006) The chromatin remodelling complex WSTF-SNF2h interacts with nuclear myosin 1 and has a role in RNA polymerase I transcription. EMBO Rep 7: 525–530. 16514417 10.1038/sj.embor.7400657 PMC1479564

[69] Jansen RP, Hurt EC, Kern H, Lehtonen H, Carmo-Fonseca M, et al (1991) Evolutionary conservation of the human nucleolar protein fibrillarin and its functional expression in yeast. J Cell Biol 113: 715–729. 2026646 10.1083/jcb.113.4.715 PMC2288999

[70] Dimitrova DS, Gilbert DM (1999) The spatial position and replication timing of chromosomal domains are both established in early G1 phase. Mol Cell 4: 983–993. 10635323 10.1016/s1097-2765(00)80227-0

[71] Chadwick BP, Willard HF (2002) Cell cycle-dependent localization of macroH2A in chromatin of the inactive X chromosome. J Cell Biol 157: 1113–1123. 12082075 10.1083/jcb.200112074 PMC2173542

[72] Kaufman PD, Kobayashi R, Kessler N, Stillman B (1995) The p150 and p60 subunits of chromatin assembly factor I: a molecular link between newly synthesized histones and DNA replication. Cell 81: 1105–1114. 7600578 10.1016/s0092-8674(05)80015-7

[73] Prelich G, Stillman B (1988) Coordinated leading and lagging strand synthesis during SV40 DNA replication in vitro requires PCNA. Cell 53: 117–126. 2894900 10.1016/0092-8674(88)90493-x

[74] Zhang Z, Shibahara K, Stillman B (2000) PCNA connects DNA replication to epigenetic inheritance in yeast. Nature 408: 221–225. 11089978 10.1038/35041601

[75] Hendzel MJ, Wei Y, Mancini MA, Hooser AV, Ranalli T, et al (1997) Mitosis-specific phosphorylation of histone H3 intiates primarily within pericentromeric heterochromatin during G2 and spreads in an ordered fashion coincident with mitotic chromosome condensation. Chromosoma 106: 348–360. 9362543 10.1007/s004120050256

[76] Hayashi-Takanaka Y, Yamagata K, Nozaki N, Kimura H (2009) Visualizing histone modifications in living cells: spatiotemporal dynamics of H3 phosphorylation during interphase. J Cell Biol 187: 781–790. 19995936 10.1083/jcb.200904137 PMC2806314

[77] Saunders WS, Chue C, Goebl M, Craig C, Clark RF, et al (1993) Molecular cloning of a human homologue of Drosophila heterochromatin protein HP1 using anti-centromere autoantibodies with anti-chromo specificity. J Cell Sci 104: 573–582. 8505380 10.1242/jcs.104.2.573

[78] Ye Q, Worman HJ (1996) Interaction between an integral protein of the nuclear envelope inner membrane and human chromodomain proteins homologous to Drosophila HP1. J Biol Chem 271: 14653–14656. 8663349 10.1074/jbc.271.25.14653

[79] Chadwick BP, Willard HF (2004) Multiple spatially distinct types of facultative heterochromatin on the human inactive X chromosome. Proc Natl Acad Sci USA 101: 17450–17455. 15574503 10.1073/pnas.0408021101 PMC534659

[80] Boggs BA, Connors B, Sobel RE, Chinault AC, Allis CD (1996) Reduced levels of histone H3 acetylation on the inactive X chromosome in human females. Chromosoma 105: 303–309. 8939823 10.1007/BF02524648

[81] Kouzarides T (2007) Chromatin modifications and their function. Cell 128: 693–705. 17320507 10.1016/j.cell.2007.02.005

[82] Chadwick BP (2007) Variation in Xi chromatin organization and correlation of the H3K27me3 chromatin territories to transcribed sequences by microarray analysis. Chromosoma 116: 147–157. 17103221 10.1007/s00412-006-0085-1

[83] Ashe A, Morgan DK, Whitelaw NC, Bruxner TJ, Vickaryous NK, et al (2008) A genome-wide screen for modifiers of transgene variegation identifies genes with critical roles in development. Genome Biol 9: R182. 19099580 10.1186/gb-2008-9-12-r182 PMC2646286

[84] Paull TT, Rogakou EP, Yamazaki V, Kirchgessner CU, Gellert M, et al (2000) A critical role for histone H2AX in recruitment of repair factors to nuclear foci after DNA damage. Curr Biol 10: 886–895. 10959836 10.1016/s0960-9822(00)00610-2

[85] Ayoub N, Jeyasekharan AD, Bernal JA, Venkitaraman AR (2008) HP1-b mobilization promotes chromatin changes that initiate the DNA damage response. Nature 453: 682–686. 18438399 10.1038/nature06875

[86] Luijsterburg MS, Dinant C, Lans H, Stap J, Wiernasz E, et al (2009) Heterochromatin protein 1 is recruited to various types of DNA damage. J Cell Bio 185: 577–586. 19451271 10.1083/jcb.200810035 PMC2711568

[87] Yuan X, Feng W, Imhof A, Grummt I, Zhou Y (2007) Activation of RNA polymerase I transcription by Cockayne Syndrome group B protein and histone methyltransferase G9a. Mol Cell 27: 585–595. 17707230 10.1016/j.molcel.2007.06.021

[88] Harnicarova Horakova A, Bartova E, Galiova G, Uhlirova R, Matula P, et al (2010) SUV39h-independent association of HP1b with fibrillarin-positive nucleolar regions. Chromasoma 119: 227–241. 10.1007/s00412-009-0252-2 20033197

[89] Zhang LF, Huynh KD, Lee JT (2007) Perinucleolar targeting of the inactive X during S phase: evidence for a role in the maintenance of silencing. Cell 129: 693–706. 17512404 10.1016/j.cell.2007.03.036

[90] Cook PJ, Ju BG, Telese F, Wang X, Glass CK, et al (2009) Tyrosine dephosphorylation of H2AX modulates apoptosis and survival decisions. Nature 458: 591–596. 19234442 10.1038/nature07849 PMC2692521

