# Supplementary material for: The WSTF-ISWI Chromatin Remodeling Complex Transiently Associates with the Human Inactive X Chromosome during Late S-Phase Prior to BRCA1 and γ-H2AX
Source: PLoS One. 2012 Nov 14;7(11):e50023. doi: 10.1371/journal.pone.0050023 (PMC3498190; doi:10.1371/journal.pone.0050023)
Supplement: Table S1 — List of BAC clone pools used for FISH with WSTF indirect-immunofluorescence. BAC clone members of each pool (1–6 as indicated in Figure 7) are highlighted in different colors and their coordinates on the X (where known) are given based on hg18, as are genes or genomic features of interest in the corresponding BAC. The BAC clone RP11-423G05 that is shown overlapping WSTF in Figure 10 is highlighted in black. (PDF) [file pone.0050023.s001.pdf]

X chromosome BACs

Pool	BAC	Start*	Finish	Genes
1	RP11-102P23	80307324	80471160	SH3BGRL
	RP11-73009	84981302	85164535	CHM
	RP11-471G13	94723861	94900251	None
	RP11-358K18	94891268	95033143	None
	RP11-402K9	99210925	99386056	None
	RP11-99E24	99373843	99554021	PCDH19
2	RP11-643F6	99643781	99890275	TNMD, TSPAN6 + SYTL4
	RP11-794O23	100121535	100322783	TRMT2B, TMEM35 + CENPI
	RP11-192G13	100579944	100766192	ARMCX4, ARMCX1 + ARMC6
	RP11-353J17	101454691	101642126	NXF2B
	RP11-398N9	102643087	102806818	MORF4L2
	RP11-541I12	103373831	103557164	ESX1
	RP11-647M7	105695984	105872849	CXORF57 + RNF128
	RP11-150F24	105832705	106002508	RNF128 + TBC1D8B
3	RP11-425O12	109203067	109404393	AMMECR1
	RP11-12D18	~109140001	109290000	TMEM164
	RP11-733H21	109968123	110073451	PAK3
	RP11-1066D24	111533817	111631587	ZCCHC16
	RP11-485D13	113641141	113824886	HTR2C
4	RP11-268A15	114639781	114792452	PLS3
	2272M5	114		DXZ4
	RP11-761E20	114943586	115133311	DXZ4 distal
	RP11-517D11	115089412	115231372	AGTR2
	RP11-27G9	115938027	116104434	None
	RP11-197B12	117211298	117315606	None
**	RP11-423G5	117448177	117632053	DOCK11
	RP11-166C19	119		CT47
	RP11-100L19	122926111	123093111	STAG2
	RP11-13E5	124998007	125165297	WDR40C
5	RP11-388O17	129		SUHW3
	RP11-754H22	130636029	130790513	X130 array
	RP11-274K13	134872885	135024305	MMGT1 + SLC9A6
	RP11-535K18	135051131	135233538	FHL1, MAP7D3 + GPR112
6	82404	153		H2A.Bbd & F8c
	1047B3	153		H2A.Bbd & F8c
	968D14	154		H2A.Bbd
	999H8	154		H2A.Bbd
	905N17	154		H2A.Bbd
	933C9	154		H2A.Bbd

*Coordinates based on hg18. Precise start and finish base pairing given for those BACs that are sequenced. Coordinates of non-sequenced BACs are based on FISH data and gene content.

**DOCK11 containing BAC clone that frequently overlapped with WSTF enrichment at the Xi.
